# Risk Factors and Patterns of Recurrence in Stage III Perforated Colorectal Cancer: A Single-Center, Retrospective, Observational Study

**DOI:** 10.7759/cureus.77446

**Published:** 2025-01-14

**Authors:** Hiroshi Asano, Yuusuke Fusejima, Makoto Takagi, Tetsuyoshi Takayama, Masaomi Suzuki

**Affiliations:** 1 General Surgery, Saitama Medical University, Moroyama, JPN

**Keywords:** carcinoma, colorectal perforation, lymphatic metastasis, r0 surgery, recurrence

## Abstract

Background: Colorectal perforation generally results in a poor prognosis with a high mortality rate. Malignant colorectal perforation may result in cancer recurrence; however, the reason for higher recurrence rates in perforated than in nonperforated colorectal cancer is unclear. Therefore, we aimed to identify factors influencing stage III perforated colorectal cancer recurrence after a microscopically margin-negative resection (R0) surgery.

Materials and methods: This single-center, retrospective, observational study enrolled patients with stage III colorectal cancer who had undergone R0 surgery between 2007 and 2019. The clinicopathological characteristics and recurrence patterns of patients with perforated (n = 34) versus nonperforated tumors (n = 120) were compared.

Results: The T4 disease proportion was significantly higher, and lymphatic invasion was more severe in the perforated group than in the nonperforated group. Significantly more dissected lymph nodes (n = 17) were observed in the nonperforated group than in the perforated group (n = 11). The rates of postoperative Clavien-Dindo III or higher complications and in-hospital mortality were significantly higher in the perforated group. Of the 23 and 96 patients who underwent long-term follow-up in the perforated and nonperforated groups, recurrence occurred in 14 (61%) and 34 patients (35%), respectively. The proportion of stage IIIC lesions was higher in the recurrence subset of the nonperforated group; however, clinicopathological characteristics did not differ significantly between the subsets of the perforated group.

Conclusions: The higher recurrence rate of stage III perforated colorectal cancer is likely due to higher T classification, lymphatic invasion, and increased lymph node metastases. Factors leading to perforation are likely related to advanced cancer stage.

## Introduction

Colorectal perforation has a high mortality rate, ranging from 12% to 26%, as it can easily progress to sepsis, disseminated intravascular coagulation, and multiple organ failure [1-3]. Despite advancements in surgical techniques and intensive care, this condition continues to have a poor prognosis. Perforation can result from various causes, with benign conditions accounting for more than half the cases. When the acute phase is effectively managed, long-term survival is possible. Nevertheless, in the case of colorectal cancer perforation, which is malignant, it is necessary to consider the potential for cancer recurrence in the long term.

Various risk factors for colorectal cancer recurrence have been identified, including lymphatic invasion, vascular invasion, pT4 staging, and clinical symptoms such as intestinal obstruction or perforation [4,5]. Previous studies have reported that perforated colorectal cancer has a higher recurrence rate compared to nonperforated colorectal cancer [6-8]. This may be due to the reduction in the scope of dissection to prioritize survival [9,10] and the dispersion of cancer cells resulting from perforation [11,12]. Some studies have reported that distant recurrence is common in perforated colorectal cancer [7,9], similar to that in typical colorectal cancer, whereas one study found a higher frequency of local recurrence in perforated colorectal cancer [11]. Although the recurrence rate of perforated colorectal cancer is notably higher than that of nonperforated colorectal cancer, the mechanisms driving this increase remain unclear.

The recurrence rate of colorectal cancer varies greatly depending on lymph node metastasis, necessitating stage-specific analysis. In a previous study, we investigated stage II perforated colorectal cancer and discovered a considerably higher rate of local recurrence compared to nonperforated colorectal cancer. In stage II, the recurrence rate is typically higher for perforated colorectal cancer than for nonperforated colorectal cancer, but the pattern of recurrence is different. This may be due to frequent T4 involvement with serosal invasion in perforated colorectal cancer. Additionally, local recurrence increases with cancer exposure, and perforation itself does not impact recurrence; however, the high recurrence rate is likely attributed to the advanced nature of cancer, which leads to perforation [13]. It is currently unknown whether this trend is similarly observed in stage III, which involves lymph node metastasis. The objective of this study was to identify specific clinicopathological factors influencing recurrence in stage III perforated colorectal cancer and compare these factors between patients with perforated and nonperforated tumors following a microscopically margin-negative resection (R0) surgery.

## Materials and methods

Study design and population

This retrospective observational study included patients with colorectal cancer who underwent surgery at the Department of General Surgery, Saitama Medical School, between January 2007 and December 2019. From this cohort, patients with and without perforation were selected from a dataset of patients who had undergone R0 surgery and were pathologically diagnosed postoperatively with stage III colorectal cancer. The presence of perforation, free gas in the abdominal cavity, and abscess formation around the intestinal tract were evaluated using computed tomography (CT) and intraoperative findings. The image assessment was performed by a surgical specialist or radiologist. Besides emergency surgery, patients who underwent preoperative percutaneous drainage or antibiotic administration followed by elective surgery were also included in the perforated group. Patients with iatrogenic perforations associated with stent placement were excluded. Patients without findings for suspicious perforation on preoperative examination or those in whom perforation occurred due to intraoperative manipulation, resulting in leakage of the intestinal contents, were included in the nonperforated group. Furthermore, patients with a history of intestinal obstruction or those who had undergone decompression procedures, such as stent implantation or colostomy for passage obstruction, were excluded from this study.

Clinicopathological background characteristics

Data on age, sex, tumor localization, depth of invasion, lymphatic invasion, venous invasion, histological type, number of dissected lymph nodes, recurrence status, and recurrence site were extracted from the patients' medical records. The clinicopathological characteristics, including recurrence information, were compared between participants with and without perforation. The Union for International Cancer Control (UICC) TNM system [14] was used for cancer stage classification. R0 was defined as the macroscopic absence of residual cancer based on the intraoperative findings, with a resection edge or ablation surface that was pathologically negative for cancer.

Follow-up

Long-term outcomes were analyzed for all patients, excluding cases of in-hospital death. Following discharge, patients were monitored at our hospital for a minimum of three years to confirm death or assess for recurrence. In the perforated group, patients were provided the option to undergo postoperative adjuvant chemotherapy after explaining the expected adverse effects due to the high risk of recurrence in patients with T4 disease, poorly differentiated adenocarcinoma, venous invasion, lymphatic invasion, and dissection of fewer than 12 lymph nodes. The adjuvant chemotherapy regimen included oral capecitabine (1,000 mg/m2 twice daily for two weeks in a three‐week cycle), oral capecitabin plus oxaliplatin (130 mg/m3 on day one in a three-week cycle), or tegafur/uracil with oral leucovorin (500 mg/day tegafur/uracil + 75 mg/day oral leucovorin, administered over four weeks followed by one week of rest) for a total of six months. Postoperative monitoring included carcinoembryonic antigen (CEA) and cancer antigen 19-9 (CA19-9) measurements, along with thoracoabdominal pelvic CT scans every six months.

Additionally, a colonoscopy was performed once annually. Postoperative follow-up was conducted for five years and was discontinued if there were no signs of recurrence. Recurrence was determined using CT images and lower gastrointestinal endoscopy; histological confirmation was not required. The test date was denoted as the date of recurrence. Local recurrence was defined as the appearance of a lesion in the peritoneum, soft tissue near the anastomosis, or at the site where the tumor originally existed. Peritoneal recurrence was defined as disseminated lesions in sites distant from the original tumor site.

Statistical analyses

All statistical analyses were conducted using BellCurve for Excel (Social Survey Research Information Co., Ltd, Tokyo, Japan). Categorical variables, including sex, tumor localization, depth of invasion, lymphatic invasion factor, venous invasion factor, histological type, and presence or absence of recurrence, were summarized as counts and percentages. Quantitative variables, such as age and number of lymph nodes dissected, were presented as medians with interquartile ranges. Chi-square tests were used to assess the associations between categorical variables.

The Mann-Whitney U test was used to analyze continuous quantitative variables, such as age and number of dissected lymph nodes. This nonparametric test was selected due to the nonnormal distribution of these variables. The Mann-Whitney U test is robust against deviations from normality and is appropriate for comparing two independent groups when the assumption of normality is not met.

Survival curves for the postoperative recurrence-free period were generated using the Kaplan-Meier method, and statistical differences between groups were analyzed using the log-rank test. In the analysis of the recurrence-free period, events were defined as either death or recurrence. A p-value of <0.05 was considered statistically significant.

Ethical approval and consent to participate

All procedures in this study involving human participants were conducted in accordance with the ethical standards of the institutional or national research committee and the 1964 Declaration of Helsinki, including its later amendments or comparable ethical standards. The Institutional Review Board of Saitama Medical University Hospital (No. 19071) approved this study and waived the requirement for written informed consent due to its retrospective design.

## Results

Study population

**Figure 1:**
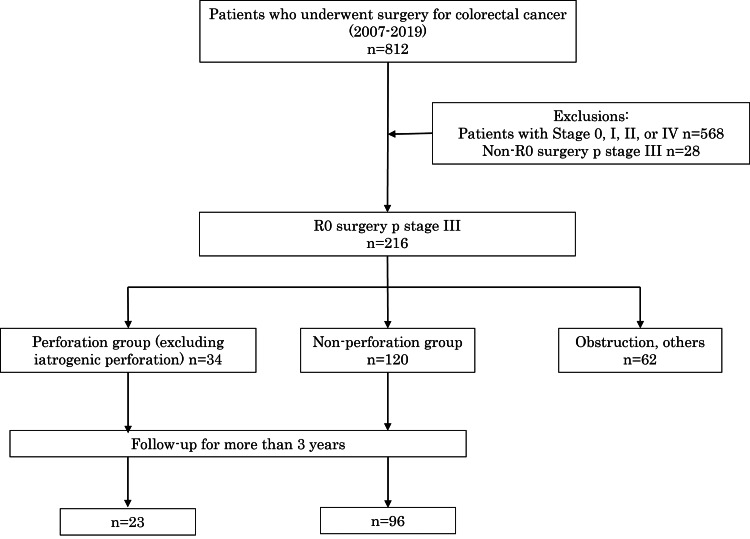
Flowchart showing the selection and classification of the study participants into experimental groups

A total of 812 patients underwent colorectal cancer surgery at our institution between 2007 and 2019. Of these, 216 patients underwent R0 surgery and were pathologically diagnosed with stage III disease with lymph node metastasis. Sixty-two patients with intestinal obstruction from a tumor or iatrogenic perforation due to preoperative endoscopic stent placement were excluded. Thirty-four patients with free gas or abscess formation on preoperative abdominopelvic CT were classified as the perforated group, whereas 120 patients who underwent elective surgery without any findings of perforation or intestinal obstruction were classified as the nonperforated group. Furthermore, postoperative follow-up was conducted for 23 patients in the perforated group and 96 in the nonperforated group (Figure 1)

Clinicopathological background characteristics

The median age of patients in both the perforated and nonperforated groups was 74 years, with both groups predominantly comprising men. The proportion of left-sided colon cancer was considerably higher in the perforated group (88%) compared to that in the nonperforated group (48%). Additionally, the proportion of T4 lesions was remarkably higher in the perforated group (44%) than in the nonperforated group (26%). Lymphatic invasion occurred in 76% of the perforated group, which was markedly higher than that in the nonperforated group (54%); however, no significant differences were observed between the two groups with respect to venous invasion and degree of differentiation. The number of dissected lymph nodes in the nonperforated group (17) was remarkably higher than that in the perforated group (11). The frequency of postoperative complications with a Clavien-Dindo grade of III or higher (38% vs. 6%, p < 0.001) and the in-hospital mortality rate (12% vs. 1%, p < 0.001) were significantly higher in the perforated group than those in the nonperforated group (Table 1).

**Table 1:** Comparison of patients’ clinicopathological characteristics (a) Chi-square test, (b) Mann-Whitney U test Categorical variables are expressed as numbers and percentages. Continuous variables are presented as medians with interquartile ranges. p-value < 0.05 was considered significant (Chi-square test or nonparametric Mann-Whitney U test)

Parameters		Perforated n = 34	Nonperforated n = 120	X^2^	p-value
Median age (years)		74 (63-81)	74 (67-79)		0.99^b^
Sex	Male	18 (53%)	67 (56%)	0.09	0.77^a^
	Female	16 (47%)	53 (44%)		
Localization	Cecum	3 (9%)	24 (20%)	22.92	<0.001^a^
	Ascending	0	24 (20%)		
	Transverse	1 (3%)	15 (13%)		
	Descending	3 (9%)	1 (1%)		
	Sigmoid	13 (38%)	29 (24%)		
	Rectum	14 (41%)	27 (23%)		
Pathological T stage	T1-3	19 (56%)	94 (78%)	4.63	0.032^a^
	T4	15 (44%)	26 (22%)		
Lymphatic invasion	No	8 (24%)	55 (46%)	5.45	0.020^a^
	Yes	26 (76%)	65 (54%)		
Venous invasion	No	8 (24%)	41 (34%)	2.00	0.16^a^
	Yes	26 (76%)	79 (66%)		
Differentiation grade	Well or moderate	30 (88%)	100 (83%)	0.48	0.49^a^
	Others	4 (12%)	20 (17%)		
Number of lymph nodes dissected		11 (7-13)	17 (10-22)		0.001^b^
Pathological stage	IIIAB	23 (68%)	96 (80%)	2.30	0.13^a^
	IIIC	11 (32%)	24 (20%)		
Clavien-Dindo	0-II	21 (62%)	113 (94%)	24.36	<0.001^a^
	III-IV	13 (38%)	7 (6%)		
In-hospital mortality		4 (12%)	1 (1%)	5.63	0.058^a^

Long-term results

Overall, 23 patients in the perforated group and 96 in the nonperforated group underwent follow-up for three years or longer after surgery. Postoperative adjuvant chemotherapy was administered to 15 patients (65%) in the perforated group and 55 patients (57%) in the nonperforated group. Recurrence was confirmed in 14 patients (61%) in the perforated group, but no significant differences were observed in background factors with respect to the presence or absence of recurrence. Recurrence was confirmed in 34 patients (35%) in the nonperforated group, and although the proportion of recurrence was markedly higher in patients with stage IIIC lesions, no significant differences were observed in other factors (Table 2).

**Table 2:** Comparisons of prognostic variables for recurrence (a) Chi-square test, (b) Mann-Whitney U test Categorical variables are expressed as numbers and percentages. Continuous variables are presented as medians with interquartile ranges. p-value < 0.05 was considered significant (Chi-square test or nonparametric Mann-Whitney U test)

Parameters		Perforated n = 23				Nonperforated n = 96			
		Recurrence, n = 14	No recurrence, n = 9	X^2^	p-value	Recurrence, n = 34	No recurrence, n = 62	X^2^	p-value
Median age (years)		72 (62-81)	66 (62-73)		0.28^b^	73 (68-78)	73 (66-78)		0.74^b^
Sex	Male	7 (50%)	4 (44%)	0.068	0.80^a^	20 (59%)	32 (52%)	2.54	0.50^a^
	Female	7 (50%)	5 (56%)			14 (41%)	30 (48%)		
Pathological T stage	T3	6 (43%)	6 (67%)	1.24	0.27^a^	24 (71%)	50 (81%)	1.26	0.26^a^
	T4	8 (57%)	3 (33%)			10 (29%)	12 (19%)		
Lymphatic invasion	No	3 (21%)	2 (22%)	0.002	0.96^a^	15 (44%)	32 (52%)	0.49	0.48^a^
	Yes	11 (79%)	7 (78%)			19 (56%)	30 (48%)		
Venous invasion	No	2 (14%)	2 (22%)	0.24	0.62^a^	8 (24%)	22 (35%)	1.46	0.23^a^
	Yes	12 (86%)	7 (78%)			26 (76%)	40 (65%)		
Differentiation grade	Well/moderate	12 (86%)	8 (89%)	0.049	0.83^a^	29 (85%)	52 (84%)	0.034	0.85^a^
	Others	2 (14%)	1 (11%)			5 (15%)	10 (16%)		
Number of lymph nodes dissected		12 (9-14)	11 (9-12)		0.57^b^	17 (11-24)	18 (10-22)		0.85^b^
Pathological stage	IIIAB	8 (57%)	6 (67%)	0.21	0.65^a^	22 (65%)	53 (85%)	5.55	0.02^a^
	IIIC	6 (43%)	3 (33%)			12 (35%)	9 (15%)		
Clavien-Dindo	0-II	10 (71%)	6 (67%)	0.059	0.81^a^	31 (91%)	59 (95%)	0.60	0.44^a^
	III-IV	4 (29%)	3 (33%)			3 (9%)	3 (5%)		
Adjuvant chemotherapy	Yes	8 (57%)	7 (78%)	1.028	0.31^a^	15 (44%)	40 (65%)	3.73	0.053^a^
	No	6 (43%)	2 (22%)			19 (56%)	22 (35%)		

**Figure 2:**
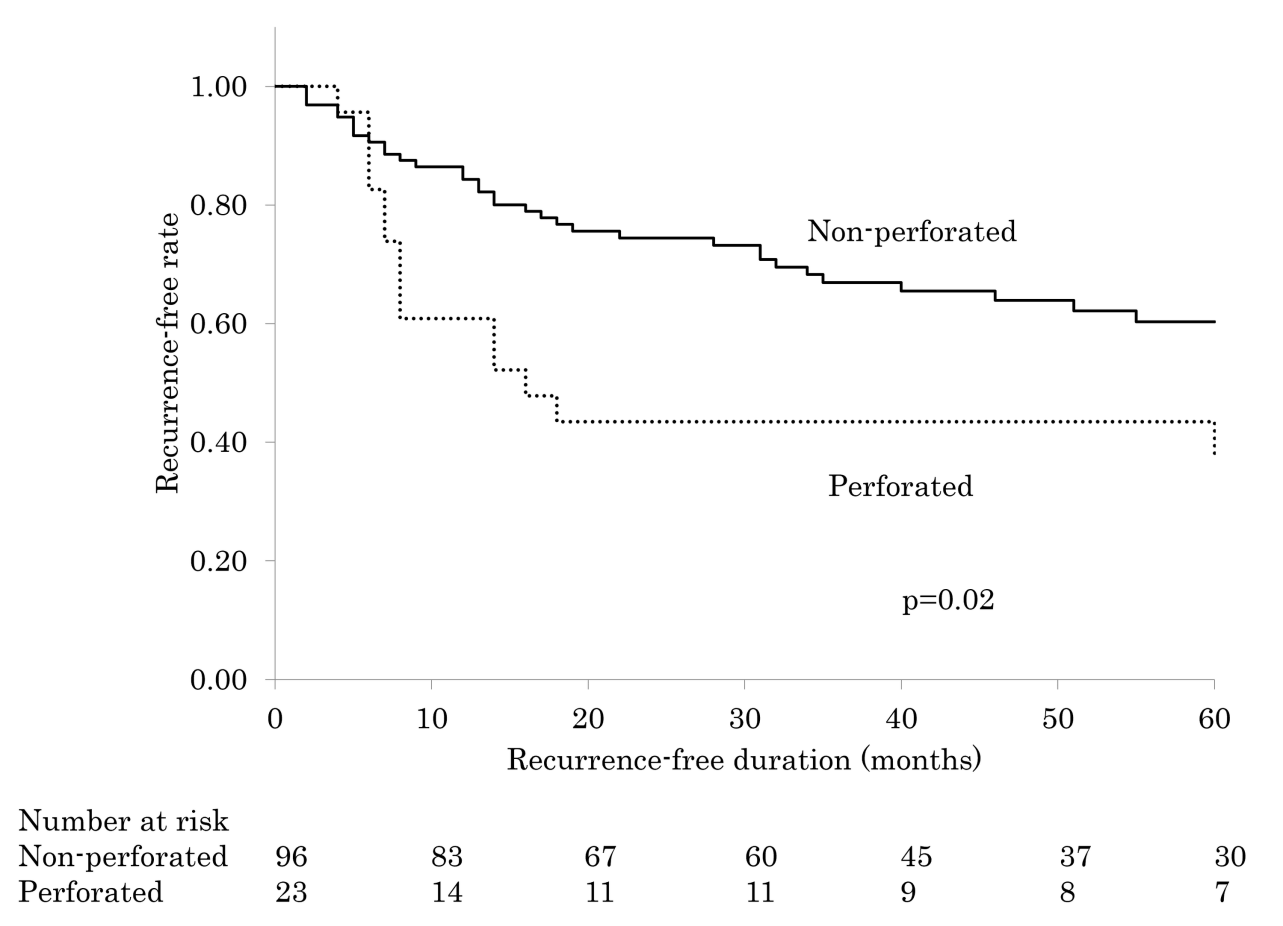
Recurrence-free curves for the perforated and nonperforated groups of patients who underwent postoperative follow-up

The average recurrence-free period after surgery was 31 months for the perforated group and 44 months for the nonperforated group. Additionally, the recurrence-free rates at three years postsurgery were 43% and 74% in the perforated and nonperforated groups, respectively. In the perforated group, 13 out of 14 patients (93%) experienced a recurrence within two years, compared to 24 out of 34 patients (71%) in the nonperforated group (Figure 2).

Recurrence pattern

In the perforated group, recurrence was observed in 14 patients and distant recurrence in 11 patients (79%). Although there were cases of multiple organ recurrences, the liver was the most common site of recurrence (n = 11), while pulmonary metastasis occurred in only one patient. Local recurrence, peritoneal dissemination, and lymph node recurrence occurred in two patients (14%) each. In the nonperforated group, distant metastasis occurred in 22 patients (65%), with the most common organ of recurrence being the liver (n = 15, 44%), followed by the lung (n = 5), bone (n = 1), and brain (n = 1). Six patients (18%) experienced local recurrence, four (12%) had peritoneal recurrence, and three (9%) experienced lymph node metastasis (Table 3).

**Table 3:** Recurrence patterns Some of the patients in this table overlapped between the two groups, indicating that they experienced more than one of these outcomes. p-value < 0.05 was considered significant (Chi-square test)

Parameters	Perforated n = 14	Nonperforated n=34	X^2^	p-value
Recurrence rate	61%	35%	4.99	0.025
Location of recurrence				
Liver	11 (79%)	15 (44%)	4.74	0.029
Lung	1 (7%)	5 (15%)	0.52	0.47
Local	2 (14%)	6 (18%)	0.081	0.78
Peritoneum	2 (14%)	4 (12%)	0.058	0.81
Node	2 (14%)	3 (9%)	0.32	0.57
Others	0 (0%)	2 (6%)	0.86	0.35

## Discussion

Previous studies have reported a higher recurrence rate in perforated colorectal cancer than in nonperforated colorectal cancer [6-8]. The present study, focused on patients with stage III disease, supports this observation. In this study, the recurrence rate for nonperforated colorectal cancer was 35%, whereas that for perforated colorectal cancer was as high as 61%. Although some studies report hematogenous metastasis, especially to the liver, as a prevalent recurrence pattern in perforated colorectal cancer [7,9], others highlight a higher incidence of local recurrence, creating a lack of consensus in the literature [11]. One study reported that local or peritoneal recurrence was more common than distant metastasis (14% vs. 86%) in stage II perforated colorectal cancer [13]; however, the current study, which was limited to stage III lesions, found that hematogenous metastasis was the predominant form of recurrence in perforated colorectal cancer, similar to that in nonperforated colorectal cancer.

Factors contributing to the high recurrence rate of perforated colorectal cancer include the dissemination of cancer cells due to perforation [11,12] and the necessity of reducing the scope of surgery to prioritize survival [15-17]. Previous studies have suggested that anastomotic leakage during colorectal cancer surgery, which can lead to the adhesion of cancer cells due to peritoneal inflammation, may increase the risk of recurrence [18,19]. Considering this mechanism, previous studies hypothesized that peritonitis caused by colorectal cancer perforation would also promote the adhesion of cancer cells and contribute to the high recurrence rate [20,21]. In this study, we did not examine the relationship between perforation type and recurrence. However, previous studies have shown that the recurrence rate is the same for cancerous perforation and perforation on the proximal side. Furthermore, there is an increased incidence of local recurrence in cases involving generalized peritonitis [22,23]. Additionally, to minimize surgical invasion and reduce the risk of further complications in patients with peritonitis, the extent of surgery is often intentionally limited, such as by reducing the extent of lymph node dissection, possibly leading to cancer recurrence due to residual cancer cells. Although these studies imply that the type of recurrence of perforated colorectal cancer is local or intraperitoneal due to peritoneal dissemination, our study shows that distant and liver recurrences were more prevalent in perforated colorectal cancer than in nonperforated cases.

The clinicopathological characteristics of perforated colorectal cancer include a markedly higher T classification, severe lymphatic invasion, and lymph node metastasis compared to those in nonperforated cases. These elements are considered risk factors for recurrence [24,25] and may be specifically associated with the recurrence of perforated lesions. If factors such as T4 disease, lymphatic invasion, and extensive lymph node metastases, rather than perforation, affect the recurrence rate of perforated colorectal cancer, we can infer that the progression of cancer may be the cause of the high recurrence rate. This may also explain the prevalence of hematogenous metastasis in colorectal cancer.

Stage III perforated colorectal cancer often recurs within a short period, with most cases occurring within two years. A similar trend is observed in stage II perforated colorectal cancer [13]. It is likely that cancer cells have already metastasized to distant organs but remain undetectable at the time of surgery. Furthermore, the disease-free interval of colorectal cancer generally shortens as the cancer progresses [26], and a shorter disease-free interval may be associated with a poorer prognosis [27]. Therefore, perforation of colorectal cancer may be related to disease progression.

In this study, the number of dissected lymph nodes was considerably lower in the perforated group than in the nonperforated group. The association between a limited number of dissected lymph nodes and an increased risk of recurrence [28,29] suggests that reducing the extent of dissection may contribute to an increase in the recurrence rate. For example, Gately et al. [30] discovered that liver recurrence is the primary form of recurrence in stage I, II, and III colorectal cancer; however, as the stage advances, the recurrence rate decreases, whereas lymph node recurrence remarkably increases. Given that stage III disease is accompanied by lymph node metastasis, the likelihood of cancer cells in the lymph is high. Nevertheless, they reported no significant differences in the number of dissected lymph nodes between the recurrent and nonrecurrent subsets of the perforated and nonperforated groups, nor could they clarify whether the reduction in the scope of lymph node dissection directly influenced the recurrence rate.

In the present study, postoperative adjuvant chemotherapy was administered to 15 of 23 patients in the perforated group for whom follow-up observation was possible. Although a greater proportion of patients in the perforated group received adjuvant chemotherapy than those in the nonperforated group, this difference was not statistically significant. Furthermore, while the effect of adjuvant chemotherapy on postoperative recurrence was not statistically significant, the recurrence rate was lower in the group receiving chemotherapy. Thus, performing standard surgery for perforated colorectal cancer, including lymph node dissection, and introducing postoperative adjuvant chemotherapy akin to that for nonperforated colorectal cancer is suggested.

The limited sample size may restrict the generalizability of our findings, highlighting the need for larger studies to validate these results. Since the study was limited to patients with stage III disease, only 34 patients with perforated colorectal cancer were enrolled over a 10-year period, with 23 undergoing follow-up for three years or longer. Generally, patients with colorectal cancer undergo follow-up observation for five years postoperatively to detect recurrence; however, some patients may discontinue hospital visits or decline follow-up observation. Additionally, follow-up may not be possible for some patients. In this study, 24 of 120 patients with nonperforated colorectal cancer and 11 of 34 patients with perforated colorectal cancer were lost to follow-up. Moreover, several patients with perforated colorectal cancer were older, and their activities of daily living extensively deteriorated during hospitalization, making it difficult for them to be discharged, thereby prolonging hospitalization. Since perforated colorectal cancer is a rare condition, further research is needed to elucidate the factors contributing to its high recurrence rate. Prospective studies are crucial to better characterize the mechanisms of recurrence, including the role of lymphatic invasion, surgical dissection limitations, and the effects of peritoneal inflammation on cancer cell dissemination.

## Conclusions

This study demonstrates that stage III perforated colorectal cancer exhibits a considerably higher recurrence rate than nonperforated colorectal cancer due to the features of more advanced disease characteristics, such as higher T classification, lymphatic invasion, and extensive lymph node metastases. These factors play a role in the high recurrence rate and are implicated in the pathogenesis of perforation. Although current data are insufficient to identify the specific factors that influence the recurrence rate, our findings support the involvement of these advanced disease components.

The prospects for future research suggest that the higher recurrence rate may be attributable to the advanced nature of perforated colorectal cancer itself. Subsequent studies should be aimed at clarifying the relationship between cancer progression and perforation in detail and elucidating how these factors impact recurrence. We acknowledge the elevated risk of recurrence in perforated colorectal cancer and stress the importance of comprehensive management strategies, including standard surgical procedures and postoperative adjuvant chemotherapy, to improve patient outcomes.
